# Furthering research on MAFLD: the APASL Metabolic fAtty lIver DiseasE coNsortium (MAIDEN)

**DOI:** 10.1007/s12072-023-10530-z

**Published:** 2023-04-20

**Authors:** Jacob George, George Lau, Takumi Kawaguchi, Jian-Gao Fan, Jia Ji-Dong, Fu-Sheng Wang, Manoj Kumar, Shiv Kumar Sarin, Masao Omata, Vincent Wai-Sun Wong, Mohammed Eslam

**Affiliations:** 1 Storr Liver Centre, The Westmead Institute for Medical Research, Westmead Hospital and University of Sydney, Westmead, NSW 2145 Australia; 2 Humanity and Health Clinical Trial Center, Humanity and Health Medical Group, Hong Kong Special Administrative Region, Hong Kong, China; 3 Division of Gastroenterology, Department of Medicine, Kurume University School of Medicine, Kurume, 830-0011 Japan; 4 Department of Gastroenterology, Xinhua Hospital, Shanghai Jiao Tong University School of Medicine, Shanghai, China; 5 Liver Research Center, Beijing Friendship Hospital, Capital Medical University, Beijing, 100050 China; 6 The Fifth Medical Center, PLA General Hospital, Beijing, 100039 China; 7 Department of Hepatology, Institute of Liver and Biliary Sciences, New Delhi, India; 8 Department of Gastroenterology, Yamanashi Prefectural Central Hospital, The University of Tokyo, Tokyo, Japan; 9 Department of Medicine and Therapeutics, The Chinese University of Hong Kong, Hong Kong, China

It has been more than 2 years since APASL in October 2020 became the first pan-national society to publish clinical practice guidelines for the diagnosis and management of fatty liver disease associated with metabolic dysregulation [1]. As part of this initiative, APASL adopted and endorsed the metabolic (dysfunction)-associated fatty liver disease (MAFLD) term and definition [2, 3] as the best way forward for diagnosing and dealing with this disease that is so prevalent in our region. Needless to say, the Asia Pacific will bear the brunt of the MAFLD burden and stemming from this, the adverse outcomes, both liver-related (as relevant to our Society), and non-liver-related, such as cardiovascular disease, type 2 diabetes mellitus, chronic kidney disease, and cancer.

Current epidemiological studies have consistently demonstrated that about 50% of overweight and obese adults from our region suffer from MAFLD [4], irrespective of any other concomitant liver diseases. A particular concern among those of Asian descent (in this context referring to all ethnicities not of European ancestry), is that of MAFLD that develops in the context of an ethnic-specific healthy body mass index (BMI). This population, sometimes referred to as lean MAFLD is estimated to account for about one-fifth of the Asian MAFLD population [5]. Unfortunately, despite a better metabolic profile in cross-sectional studies [6], the long-term clinical outcomes for these patients are similar to those of individuals with MAFLD who are overweight or obese [6]. Thus, clinicians caring for patients of healthy weight must be particularly mindful to assess for MAFLD in those with type 2 diabetes, and to assess for elements that suggest metabolic dysregulation as highlighted in the MAFLD definition [3]. The third group of individuals in whom MAFLD is a particular problem is patients with type 2 diabetes mellitus, considered in the diagnostic pathway as having the most severe form of systemic metabolic dysregulation. Studies from our region have consistently shown that the risk of cardiovascular risk, extrahepatic cancer, and liver-related outcomes including advanced fibrosis and hepatocellular carcinoma are the greatest among patients with MAFLD and type 2 diabetes mellitus [7, 8].

A unique and singular attribute of MAFLD is that it tells us what the disease is, and not what it is not. This ensures a homogenous population governed by the parameters of the definition and sub-categorized according to the three stratification criteria. Stemming from the definition, we now have two other distinct groups in which liver disease can be further investigated through clinical and laboratory research. The first comprises individuals with hepatic steatosis but without other requisite features of metabolic dysregulation and other causes of hepatic steatosis. The questions going forward in this group of individuals include: do they have pre-MAFLD, akin to pre-diabetes; do they have steatosis related to undeclared alcohol use or high endogenous alcohol production [9]; another cause of steatosis that has not been adequately investigated (e.g., coeliac disease, Wilson disease, liposomal acid lipase deficiency etc.), or do they have as yet some unknown cause of steatosis (idiopathic for now)? At the other end of the spectrum where MAFLD provides a distinct advantage is the ability to diagnose fatty liver due to metabolic dysregulation based on its definition, even in the context of another liver disease(s). To date, one is unable to investigate and characterize these patients more precisely in terms of liver pathology, natural history, or outcomes, since we do not know what constitutes the key attributes of MAFLD. Many published studies including from our region have shown that the disease trajectory of those with MAFLD and another liver disease whether it be related to alcohol, viral hepatitis, or autoimmune disease, is different from that of a single entity [10, 11]. Clearly, more research needs to be done both in human and animal models on the mechanisms by which MAFLD impacts these diseases and to answer the question as to whether it is merely an additive interaction or in fact synergistic. If the latter, what are the common and distinct mechanisms from having a single disease? In many instances, the answers to such questions are not immediately apparent. For example, is the outcome of MAFLD in chronic hepatitis B with viral suppression only determined by MAFLD? or is there a persistent contribution from intrahepatic cccDNA, very low levels of virus replication or from immune responses to hepatitis B? Such questions are even more relevant when we consider liver cancer that arises in the context of MAFLD and another disease such as virally suppressed or untreated hepatitis B or for that matter, cured or untreated hepatitis C. For example, in cured hepatitis C with early-stage fibrosis, does the preceding length of virus replication set the stage for hepatocyte clonal proliferation such that hepatocellular carcinoma development and progression will be different from that of a person with MAFLD only? The research questions are only limited by ones’ scientific curiosity, all thanks to MAFLD conceptualization.

In the broader literature, 3 years since its conceptualisation, there have been several thousand papers published investigating the applicability of the new terminology for clinical practice, and direct head-to-head comparisons of non-alcoholic fatty liver disease (NAFLD) versus MAFLD in common datasets. In addition, a variety of societies across the board in all global regions have adopted the MAFLD terminology for clinical practice [12–15]. Briefly, the sum of these papers indicates that MAFLD has high relevance to clinical practice, increases disease awareness among clinicians outside of the field of gastroenterology and hepatology, and aligns better with other lifestyle-related non-communicable diseases including cardiovascular disease, chronic kidney disease, and type 2 diabetes [16–21]. The bulk of evidence indicates that MAFLD, as compared to NAFLD, better predicts advanced chronic liver disease, adverse hepatic outcomes and as well cardiovascular mortality, cancer mortality, and all-cause mortality than NAFLD [20]. Indeed, in those with hepatic steatosis that does not meet the definition of MAFLD, liver-related outcomes appear no different from that of the general population [22]. Finally, attesting to its utility, a pediatric definition for MAFLD has also been published [23]. This level of evidence and widespread acceptance could not have eventuated except through the prolific work of clinicians, patient groups and other stakeholders across the globe, something that APASL should be proud of as an “early-adopter”.

On the flip side, there have been calls to suggest that the MAFLD criteria are premature and should await the development of a specific diagnostic test similar to what we have for hepatitis B or C. The cogent question however is whether we will ever have such an easily available diagnostic test since MAFLD is the outcome of a dysregulated metabolic milieu with all its complexities. Metabolomics and proteomics are unlikely to be the answer as while they may predict metabolic dysregulation, and even if we ignore cost considerations, they are unlikely to be of sufficient diagnostic accuracy at the bedside given the time variance in the results based on dietary intake, physical activity, and other lifestyle factors.

As published over two decades ago, insulin resistance is the key pathophysiological determinant of MAFLD [24]. While more complex tests such as euglycemic hyperinsulinemic clamp studies exist for the diagnosis of insulin resistance, they are not practical at the bedside and are not specific to MAFLD. Hence, what is needed is an operational definition which best suits the needs of clinicians today and one that can be applied in all healthcare settings. We do have this, and it is MAFLD. In contrast, tests for disease staging, irrespective of etiology, are more likely to become available whether it be through imaging or technologies such as elastography, or through blood-based non-invasive tests for liver fibrosis, of which there are a plethora [25].

Another suggestion has been that only by excluding alcohol in the development of fatty liver allows for a broad differential diagnosis. The fundamental misconception here is a misunderstanding of the *intent* of a disease definition. For every disease, diagnostic criteria relate to the causative factors, and for MAFLD, the criterion is metabolic dysfunction. The presence of such dysfunction in conjunction with hepatic steatosis defines MAFLD. The presence of a second disease (e.g., alcohol-related liver disease) should in no way impact the diagnosis of the first, and the second disease is precisely that, a second disease. An analogy would be a patient who is both serum HCV RNA-positive and HBsAg-positive. The criterion for each of these diseases indicates the basis of each diagnosis and has nothing to do with any other coexistent disease. If a patient has obesity and hepatic steatosis, then irrespective of a contribution from alcohol or not, they have MAFLD. If it is deemed that a particular level of alcohol consumption does not impact the liver (criteria that should be established by those in the alcohol field), then those individuals would have MAFLD only as a single etiologic cause for their disease. If on the other hand, they have MAFLD and a contribution from alcohol, then they have precisely that—two causes for their liver disease.

One might ask, where to from here? Clearly, there is a breadth of new knowledge already that has been enabled by the conceptualisation of MAFLD, including evidence that patients in current clinical trials universally have MAFLD. However, to those in the field, this is just the tip of the iceberg of the bounties that are yet to come. How can we capitalize on this initiative for the APASL region and the world, cognizant of the fact that we will be at the epicenter of the epidemic?

To this end, APASL has established **MAIDEN (M**etabolic f**A**tty l**I**ver **D**iseas**E** co**N**sortium) comprising key opinion leaders from the Asia Pacific region. The overarching principles of APASL MAIDEN are a patient-oriented focus, transparency and equity, credible data sharing, and professional communication. The consortium in its charter will uphold and embed an evidence-based scientific approach that we expect will be acceptable to the 5 Ps (Patients, Physicians, Pharma, Policy-makers, and the Public). Among its many goals, the key aims of MAIDEN are to (a) further the science and practice of Hepatology as it pertains to MAFLD in the APASL region, (b) engage with sister societies on fatty liver disease research and clinical practice, (c) establish a forum for dialog and for research collaboration, (d) enhance the role of APASL as a key driver of fatty liver disease research in the region and globally, (e) contribute to clinical trials for patients within our region, and (f) provide educational opportunities and resources on fatty liver disease for our patients and their families, policy-makers, and healthcare providers.

The governance structure of MAIDEN includes a Chair, co-Chairs, a standing committee, together with panel members comprising key opinion leaders; it reports to the senior executive of the APASL steering committee. Thankfully, there is no dearth of patients, clinical databases, and bench-to-bedside research across the globe to provide unique opportunities for collaboration and for knowledge gain. This then is the intent of the consortium, to bring together clinicians, researchers and patient representatives to tackle the burden of MAFLD. We welcome all with such intent to join this APASL MAIDEN initiative. In the coming years, APASL MAIDEN will be the accommodative dynamo of APASL to translate science to benefit patients with MAFLD especially in the Asia Pacific region.
